# Short-term and one-year outcome of infective endocarditis in adult patients treated in a Finnish teaching hospital during 1980–2004

**DOI:** 10.1186/1471-2334-7-78

**Published:** 2007-07-17

**Authors:** Maija Heiro, Hans Helenius, Saija Hurme, Timo Savunen, Erik Engblom, Jukka Nikoskelainen, Pirkko Kotilainen

**Affiliations:** 1 Department of Medicine, Turku University Hospital, Turku, Finland; 2 Department of Biostatistics, University of Turku, Turku, Finland; 3 Department of Surgery, Turku University Hospital, Turku, Finland

## Abstract

**Background:**

Previous studies on factors predicting the prognosis of infective endocarditis have given somewhat conflicting results. Our aim was to define the factors predicting the outcome of patients treated in a Finnish teaching hospital.

**Methods:**

A total of 326 episodes of infective endocarditis in 303 patients treated during 1980–2004 were evaluated for short-term and 1-year outcome and complications.

**Results:**

Infection of 2 native valves and the occurrence of neurological complications, peripheral emboli, or heart failure significantly predicted both in-hospital and 1-year mortality, while age ≥65 years or the presence of a major criterion or vegetation on echocardiography predicted death within 1 year. A significant trend was observed between the level of serum C-reactive protein (CRP) on admission and both the short-term and 1-year outcome. In the patients who had CRP values ≥100 mg/l on admission, the hazard ratio for in-hospital death was 2.9-fold and the hazard ratio for 1-year death was 3.9-fold as compared to those with lower CRP values. Male sex and age < 64 years significantly predicted a need for both in-hospital and 1-year surgery, as did the development of heart failure or the presence of a major criterion or vegetation on echocardiography. Peripheral emboli were associated with a need for in-hospital surgery, while *Streptococcus pneumoniae *as the causative agent or infection of 2 native valves predicted a need for surgery within 1 year from admission.

**Conclusion:**

Some of the factors (e.g. heart failure, neurological complications, peripheral emboli) predicting a poor prognosis and/or need for surgery were the same observed in previous studies. A new finding was that high CRP values (≥100 mg/l) on admission significantly predicted both short-term and 1-year mortality.

## Background

Infective endocarditis is a diagnostic and therapeutic challenge to clinicians. Despite major advances in cardiac imaging technology, antimicrobial treatment and surgical techniques, the morbidity and mortality associated with infective endocarditis remains high. Several previous studies from the 1990's and 2000's show that mortality of endocarditis is still from 10% to 24% [1-6]. Some authors report a decreasing trend in mortality, and this has been attributed either to lower operative mortality [7], or technically more successful early valve surgery [6]. Previous studies have attempted to identify various clinical and microbial factors predicting either short-term or long-term mortality of infective endocarditis, with somewhat conflicting results [5,8-12].

Our previous reports on patients treated for infective endocarditis in our hospital from 1980 onwards have focused on diagnostic classification, neurological manifestations, and the utility of the serum C-reactive protein (CRP) in assessing the outcome of the disease [13-15]. We have also published the changes we have seen in the presentation of endocarditis during the past 25 years [16]. In the present study, we analyse the short-term and 1-year clinical outcome of these patients in more detail with the aim to delineate the factors predicting an adverse outcome in patients treated for endocarditis in a Finnish teaching hospital.

## Methods

The short-term and 1-year outcome of infective endocarditis was analysed in 303 patients with 326 episodes of the disease treated at the Turku University Hospital, Turku, Finland, between 1980 and 2004. The hospital is a 1000-bed teaching facility with a cardiothoracic surgical department, serving as a tertiary referral centre for the southwestern part of the country, and as a primary care facility for infectious diseases for a region of about 200.000 inhabitants.

For each patient, data on age, sex, underlying diseases, causative agents of infective endocarditis, affected valves and echocardiographic findings, as well as the development of complications were collected by us for our previous study focusing on the changes of the clinical characteristics of endocarditis during the 25-year period [16]. For the present study, the mortality and the need for surgical treatment were recorded in detail for all patients during a period of 1 year from admission. In addition to hospital records, dates of possible deaths were obtained from the National Population Registry, in which all deaths and causes of deaths of the Finnish residents are registered. CRP and erythrocyte sedimentation rate (ESR) values and white blood cell (WBC) counts on admission were also registered. The data were used to analyse the association between various patient and disease characteristics and the development of complications, mode of treatment and mortality of infective endocarditis. The factors predicting a poor short-term and 1-year clinical outcome and the requirement for surgical treatment were also delineated.

Short-term outcome was defined as the outcome during the index hospitalisation.

The study was approved by the Institutional Committee on human research.

### Statistical analysis

The associations between the clinical characteristics and cumulative mortality or cumulative need for surgery was studied using survival (or event history) analysis. First, during different time periods the cumulative percentages for death (table 1) or for need of surgery (table 2) were estimated using Kaplan-Meyer technique. Differences in cumulative percentages between groups were tested using log-rank test. Differences between groups were quantified by calculating hazard ratios using Cox's regression models (tables 4 and 5). Associations between in-hospital complications and death or need for surgery were tested using chi squared test. P-values less than 0.05 were considered as statistically significant. Statistical computations were carried out using SAS^® ^release 9.1/2005.

**Table 1:** Association between cumulative mortality within 1 year and patient characteristics in 326 episodes of infective endocarditis

	**Mortality during n (%)**								
	N	In-hospital^a^	p value	3 months	p value	6 months	p value	1 year	p value
**Sex**			0.423		0.261		0.328		0.707
Male	234	35 (15.0)		34 (14.7)		38 (16.5)		39 (17.0)	
Female	92	11 (12.0)		9 (9.9)		11 (12.1)		14 (15.7)	
**Age**			0.065		0.022		0.013		0.004
18–64 years	209	23 (11.0)		21 (10.2)		24 (11.6)		25 (12.1)	
≥65 years	117	23 (19.7)		22 (19.0)		25 (21.8)		28 (24.9)	
**Etiology**			0.031^b^		0.004^b^		0.012^b^		0.036^b^
*Staphylococcus aureus*	75	14 (18.7)		14 (18.9)		15 (20.3)		15 (20.3)	
CoNS^c^	31	6 (19.4)		6 (19.4)		6 (19.4)		6 (19.4)	
Viridans streptococci	67	10 (14.9)		8 (12.0)		11 (16.8)		12 (18.4)	
Enterococcus faecalis	25	2 (8.0)		2 (8.0)		2 (8.0)		3 (12.8)	
*Streptococcus pneumoniae*	11	5 (45.5)		5 (45.5)		5 (45.5)		5 (45.5)	
Other	28	2 (7.1)		1 (3.6)		2 (7.3)		2 (7.3)	
Negative	89	7 (7.9)		7 (8.1)		8 (9.2)		10.(11.6)	
**Affected valves**			<0.001^b^		<0.001^b^		<0.001^b^		<0.001^b^
Aortic	113	16 (14.2)		15 (13.4)		16 (14.4)		17 (15.4)	
Mitral	96	6 (6.3)		7 (7.3)		8 (8.4)		9 (9.5)	
Tricuspid	18	2 (11.1)		1 (5.9)		2 (11.8)		2 (11.8)	
Two native valves	32	14 (43.8)		13 (40.6)		14 (43.8)		15 (46.9)	
Prosthetic valve(s)	67	8 (11.9)		7 (10.6)		9 (13.7)		10 (15.3)	
**Echocardiography**									
Major criterion			0.343		0.090		0.028		0.012
Yes	221	37 (16.7)		34 (15.6)		40 (18.4)		44 (20.4)	
No	105	9 (8.6)		9 (8.7)		9 (8.7)		9 (8.7)	
Vegetation			0.097		0.047		0.010		0.003
Yes	198	36 (18.2)		32 (16.3)		38 (19.5)		42 (21.7)	
No	128	10 (7.8)		11 (8.7)		11 (8.7)		11 (8.7)	
**Neurological events^d^**			0.009		0.003		0.003		0.005
Yes	86	21 (24.4)		19 (22.5)		21 (24.9)		22 (26.1)	
No	240	25 (10.4)		24 (10.1)		28 (11.8)		31 (13.2)	
**Peripheral emboli^d^**			0.032		0.073		0.016		0.022
Yes	102	22 (22.0)		18 (18.3)		22 (22.6)		23 (23.7)	
No	224	24 (10.6)		25 (11.1)		27 (12.1)		30 (13.5)	
**Heart failure^d^**			<0.001		<0.001		<0.001		<0.001
Yes	178	41 (23.0)		38 (21.8)		44 (25.5)		47 (27.5)	
No	148	5 (3.4)		5 (3.4)		5 (3.4)		6 (4.1)	
**In-hospital surgery**			0.751		0.614		0.551		0.552
Yes	89	15 (16.9)		13 (14.7)		15 (17.0)		16 (18.2)	
No	237	31 (13.1)		30 (12.8)		34 (14.6)		37 (16.0)	
**CRP on admission^e^**			0.005		<0.001		<0.001		<0.001
≥100 mg/l	148	28 (18.9)		29 (19.7)		31 (21.1)		34 (23.3)	
< 100 mg/l	124	8 (6.5)		5 (4.1)		8 (6.6)		8 (6.6)	
**Creatinine on admission^f^**			0.075		0.017		0.052		0.160
≥100 μmol/l	108	28 (18.9)		19 (17.7)		19 (17.7)		19 (17.7)	
< 100 μmol/l	174	17 (9.8)		15 (8.7)		18 (10.4)		22 (12.8)	

^a^Mean (SD) duration of in-hospital treatment was 55 (33), range 4 to 272 days; ^b^p values for overall group differences; ^c^coagulase-negative staphylococci; ^d^within 3 months from admission; ^e^data available for 272 episodes; ^f^data available for 282 episodes

**Table 2:** Cumulative proportion of patients needing surgery within 1 year and clinical characteristics in 326 episodes of infective endocarditis

	**Need for surgery n (%)**								
	N	In-hospital^a^	p value	3 months	p value	6 months	p value	1 year	p value
**Sex**			0.002		0.006		0.010		0.011
Male	234	74 (31.6)		78 (35.0)		83 (37.6)		88 (40.2)	
Female	92	15 (16.3)		16 (18.2)		19 (22.1)		21 (24.8)	
**Age**			<0.001		<0.001		<0.001		<0.001
18–64 years	209	73 (34.9)		77 (38.1)		84 (41.9)		88 (44.1)	
≥65 years	117	16 (13.7)		17 (15.4)		18 (16.5)		21 (20.2)	
**Etiology**			0.584^b^		0.516^b^		0.1210^b^		0.147^b^
*Staphylococcus aureus*	75	17 (22.7)		19 (28.6)		19 (28.6)		20 (30.4)	
CoNS^c^	31	9 (29.0)		9 (30.6)		9 (30.6)		9 (30.6)	
Viridans streptococci	67	20 (22.9)		20 (31.1)		25 (39.9)		28 (45.4)	
Enterococcus faecalis	25	4 (160)		4 (17.4)		4 (17.4)		4 (17.4)	
*Streptococcus pneumoniae*	11	4 (36.4)		5 (49.1)		6 (61.8)		6 (61.8)	
Other	28	8 (28.6)		8 (28.6)		10 (36.8)		10 (36.8)	
Negative	89	27 (30.0)		29 (33.1)		29 (33.1)		32.(37.0)	
**Affected valves**			<0.001^b^		<0.001^b^		<0.001^b^		<0.001^b^
Aortic	113	44 (38.9)		45 (42.4)		46 (43.5)		48 (45.8)	
Mitral	96	11 (11.5)		14 (15.1)		20 (21.9)		24 (26.9)	
Tricuspid	18	0 (0)		0 (0)		0 (0)		0 (0)	
Two native v alves	32	15 (46.9)		15 (50.5)		16 (55.5)		17 (60.4)	
Prosthetic valve(s)	67	19 (28.4)		20 (30.9)		20 (30.9)		20 (30.9)	
**Echocardiography**									
Major criterion			<0.001		<0.001		<0.001		0.001
Yes	221	77 (34.8)		78 (37.1)		86 (41.6)		89 (43.3)	
No	105	12 (11.4)		16 (16.0)		16 (16.0)		20 (20.5)	
Vegetation			0.025		0.022		0.003		0.006
Yes	198	66 (33.3)		66 (35.3)		74 (40.3)		77 (42.3)	
No	128	23 (18.0)		28 (22.7)		28 (22.7)		32 (26.3)	
**Neurological events^d^**			0.431		0.253		0.247		0.149
Yes	86	28 (32.6)		28 (35.3)		30 (38.3)		33 (43.0)	
No	240	61 (25.4)		66 (28.5)		72 (31.5)		76 (33.5)	
**Peripheral emboli^d^**			0.079		0.038		0.072		0.073
Yes	102	36 (36.0)		36 (38.7)		37 (40.1)		39 (42.8)	
No	224	53 (23.5)		58 (26.7)		65 (30.2)		70 (32.9)	
**Heart failure^d^**			<0.001		<0.001		<0.001		<0.001
Yes	178	64 (36.0)		67 (40.7)		70 (43.1)		73 (45.6)	
No	148	25 (16.9)		27(18.5)		32 (22.0)		36 (25.0)	
**Intravenous drug use**			0.170		0.074		0.045		0.030
Yes	25	3 (12.0)		3 (12.6)		3 (12.6)		3 (12.6)	
No	301	86 (28.6)		91 (31.7)		99 (35.0)		106 (39.7)	
**ESR on admission^e^**			0.005		0.004		0.010		0.003
≥50 mm/h	99	18 (18.2)		19 (19.8)		22 (23.2)		23 (24.5)	
< 50 mm/h	93	31 (33.3)		35 (39.6)		36 (40.8)		40 (46.0)	

^a^Mean (SD) duration of in-hospital treatment was 55 (33), range 4 to 272 days; ^b^p values for overall group differences; ^c^coagulase-negative staphylococci; ^d^within 3 months from admission; ^e^data on erythrocyte sedimentation rate available for 192 episodes

**Table 3:** Association between in-hospital^a ^complications and clinical characteristics in 326 episodes of infective endocarditis

	N	**Neurologic events **n (%)	p value	**Cerebral emboli **n (%)	p value	**Peripheral emboli **n (%)	p value	**Heart failure **n (%)	p value
**Sex**			0.404		0.297		0.354		0.538
Male	234	65 (27.8)		38 (16.2)		77 (32.9)		125 (53.4)	
Female	92	21 (22.8)		10 (10.9)		25 (27.2)		53 (57.6)	
**Age**			0.695		1.0		0.619		0.037
18–64 years	209	57 (27.3)		31 (14.8)		63 (30.1)		105 (50.2)	
≥65 years	117	29 (24.8)		17 (14.5)		39 (33.3)		73 (62.4)	
**Etiology**			0.035^b^		0.182^b^		<0.001^c^		0.019^b^
*Staphylococcus aureus*	75	26 (34.7)		14 (18.7)		39 (52.0)		37 (49.3)	
CoNS^c^	31	7 (22.6)		5 (16.1)		3 (9.7)		20 (64.5)	
Viridans streptococci	67	18 (26.9)		15 (22.4)		20 (29.9)		28 (41.8)	
Enterococcus faecalis	25	6 (24.0)		2 (8.0)		7 (28.0)		17 (68.0)	
*Streptococcus pneumoniae*	11	7 (63.6)		2 (18.2)		3 (27.3)		10 (90.9)	
Other pathogens	28	6 (21.4)		2 (7.1)		9 (32.1)		14 (50.0)	
Negative	89	16 (18.0)		8 (9.0)		21 (23.6)		52.(58.4)	
**Affected valves**			0.170^b^		0.105^b^		<0.001^b^		0.002^b^
Aortic	113	30 (26.6)		16 (14.2)		43 (38.1)		64 (56.6)	
Mitral	96	25 (26.0)		17 (17.7)		24 (25.0)		46 (47.9)	
Tricuspid	18	3 (16.7)		0 (0)		10 (55.6)		4 (22.2)	
Two native valves	32	14 (43.8)		8 (25.0)		18 (56.3)		25 (78.1)	
Prosthetic valve(s)	67	19 (20.9)		7 (10.5)		7 (10.5)		39 (58.2)	
**Echocardiography**									
Major criterion			0.504		1.0		0.055		0.057
Yes	221	61 (27.6)		33 (14.9)		77 (34.8)		129 (58.4)	
No	105	25 (23.8)		15 (14.3)		25 (23.8)		49 (46.7)	
Vegetation			0.158		0.425		<0.001		0.088
Yes	198	58 (29.3)		32 (16.2)		76 (38.4)		116 (58.6)	
No	128	28 (21.9)		16 (12.5)		26 (20.37)		62 (48.4)	
**Intravenous drug use**			1.0		1.0		0.012		0.146
Yes	25	6 (24.0)		3 (12.0)		14 (56.0)		10 (40.0)	
No	301	80 (26.6)		45 (15.0)		88 (29.2)		168 (55.8)	

^a^Mean (SD) duration of in-hospital treatment was 55 (33), range 4 to 272 days; ^b^p values for overall group differences; ^c^coagulase-negative staphylococci

**Table 4:** Factors predicting in-hospital^a ^mortality and mortality within 1 year from admission in 326 episodes of infective endocarditis

	**Hazard ratio for in-hospital death**	95% CI	p value	**Hazard ratio for death within 1 year**	95% CI	p value
**Age**			0.068			0.005
18–64 years	1			1		
≥65 years	1.72	1.0–3.03		2.17	1.27–3.70	
**Sex**			0.426			0.707
Female	1			1		
Male	1.32	0.67–2.60		1.12	0.61–2.10	
**Etiology**			0.060^b^			0.066^b^
*Staphylococcus aureus*	1			1		
CoNS^c^	0.87	0.33–2.26		0.93	0.36–2.40	
Viridans streptococci	0.90	0.40–2.03		0.85	0.40–1.81	
*Enterococcus faecalis*	0.36	0.08–1.57		0.55	0.16–1.90	
*Streptococcus pneumoniae*	2.37	0.85–6.59		2.58	0.94–7.09	
Other pathogens	0.35	0.08–1.54		0.31	0.07–1.36	
Culture negative	0.42	0.17–1.05		0.51	0.23–1.13	
**Affected valves**			<0.001^b^			<0.001^b^
Prosthetic	1			1		
Aortic	1.13	0.48–2.65		1.01	0.46–2.19	
Mitral	0.45	0.16–1.31		0.60	0.24–1.47	
Tricuspid	1.04	0.22–4.88		0.73	0.16–3.35	
Two native valves	3.55	1.49–8.47		3.75	1 68–8.34	
**Echocardiography**						
Major Criterion			0.346			0.015
No	1			1		
Yes	1.43	0. 68–3.00		2.44	0.19–5.0	
Vegetation			0.102			0.005
No	1.			1		
Yes	1.81	0.90–3.68		2.62	1.35–5.09	
**Neurological complications^d^**			0.009			0.003
No	1			1		
Yes	2.17	1.21–3.90		2.30	1.34–3.96	
**Peripheral emboli^d^**			0.036			0.016
No	1			1		
Yes	1.86	1.04–3.33		1.95	1.14–3.35	
**Heart failure^d^**			<0.001			<0.001
No	1			1		
Yes	5.98	2.35–15.17		7.62	3.26–17.83	
**Serum CRP on admission**			0.008			<0.001
< 100 mg/l	1			1		
≥100 mg/l	2.92	1.33–6.40		3.90	1.81–8.43	

^a^Mean (SD) duration of in-hospital treatment was 55 (33), range 4 to 272 days; ^b^p values for overall group differences; ^c^coagulase-negative staphylococci; ^d^within 3 months from admission

**Table 5:** Factors predicting in-hospital^a ^surgery and surgery within 1 year from admission

	**Hazard ratio for in-hospital surgery**	95% CI	p value	**Hazard ratio for surgery within 1 year**	95% CI	p value
**Age**			<0.001			<0.001
≥65 years	1			1		
18–64 years	3.03	1.76 – 5.21		2.57	1.59 – 4.13	
**Sex**			0.003			0.013
Female	1			1		
Male	2.30	1.32 – 4.02		1.83	1.14 – 2.95	
**Etiology**			0.611^b^			0.178^b^
*Staphylococcus aureus*	1			1		
CoNS^c^	1.17	0.52 – 2.63		1.07	0.49 – 2.36	
Viridans streptococci	1.55	0.81 – 2.96		1.56	0.88 – 2.77	
*Enterococcus faecalis*	0.59	0.20 – 1.76		0.53	0.18 – 1.56	
*Streptococcus pneumoniae*	1.59	0.53 – 4.76		2.67	1.07 – 6.65	
Other pathogens	1.28	0.55 – 2.97		1.30	0.61–2.77	
Culture negative	1.38	0.75 – 2.54		1.34	0.77 –2.35	
**Affected valve**			<0.001^b^			0.002^b^
Prosthetic	1			1		
Aortic	1.41	0.82 – 2.42		1.47	0.87–2.48	
Mitral	0.30	0.14 – 0.65		0.67	0.37–1.22	
Tricuspid	NA^d^	NA		NA	NA	
Two native valves	1.77	0.90 – 3.49		2.22	1.16–4.25	
**Echocardiography**						
Major Criterion			0.004			<0.001
No	1			1		
Yes	2.99	1.63 – 5.50		2.57	1.58 – 4.18	
Vegetation			0.027			0.007
No	1.			1		
Yes	1.71	1.06 – 2.76		1.76	1.17 – 2.66	
**Neurological complications^e^**			0.399			0.184
No	1			1		
Yes	1.22	0.77 – 1.94		1.32	0.88 – 1.99	
**Peripheral emboli^e^**			<0.001			0.112
No	1			1		
Yes	6.12	3.97 – 9.42		1.37	0.93 – 2.03	
**Heart failure^e^**			<0.001			<0.001
No	1			1		
Yes	2.26	1.42 – 3.59		2.27	1.52 – 3.39	
**Serum CRP on admission**						
< 100 mg/l	1			1		
≥100 mg/l	0.64	0.40–1.03	0.063	0.72	0.47–1.09	0.121
**ESR^f ^on admission**						
< 50 mm/h	1			1	0.28–0.78	0.004
≥50 mm/h	0.44	0.25–0.79	0.006	0.47		

^a^Mean (SD) duration of in-hospital treatment was 55 (33), range 4 to 272 days; ^b^p values for overall group differences; ^c^coagulase-negative staphylococci; ^d^NA; not applicable, 0 number of cases; ^e^within 3 months from admission; ^f^erythrocyte sedimentation rate

## Results

Of the 326 episodes, 224 were designated as definite infective endocarditis and the rest as possible infective endocarditis by the Duke criteria [17]. There were 234 episodes in men and 92 episodes in women. The mean age (SD) was 54.4 (17.3) years (range 18 to 87 years). Transthoracic echocardiography (TTE) was performed in all episodes of IE, followed by transesophageal echocardiography (TEE) in 184 episodes. The characteristics of the patients are presented in tables 1 and 2.

### Short-term and 1-year outcome

#### Patient characteristics

Fifty-three patients died of infective endocarditis during 1 year from admission; 23 of them within 30 days from admission, and 20 between 1 and 3 months after admission. The cumulative mortality in various patient groups is presented in table 1. Mortality was significantly associated with age (≥65 years) except during the index hospitalisation, but not with gender. At all time points, mortality was significantly higher in *Streptococcus pneumoniae *endocarditis than in endocarditis caused by other pathogens or in culture-negative cases (all p values < 0.006), and significantly higher in infections of 2 native valves than in infections affecting only one native valve or a prosthetic valve (all p values < 0.001). At 3 months, mortality of *Staphylococcus aureus *endocarditis was significantly higher than mortality of endocarditis caused by other pathogens excluding *S. pneumoniae *(p = 0.032). At 6 months, the difference still seemed to exist, but did not reach statistical significance (p = 0.061). Mortality was significantly associated with the occurrence of neurological complications, peripheral emboli (except at 3 months), and heart failure before 3 months after admission. There were no statistically significant differences in the outcome between the patients with different neurological complications (embolic brain infarction, brain haemorrhage, transient ischaemic stroke, meningitis) (p values between 0.737 to 0.934). At 6 months and 1 year, mortality was associated with the presence of a major criterion or a vegetation on echocardiography, and at 3 months, also with the presence of a vegetation. Mortality was not significantly associated with conditions like diabetes, collagenosis, malignancy, or intravenous drug use (IVDU) (all p values > 0.065).

During the study period, mortalities were not different for the patients who were treated surgically or conservatively during the index hospitalisation (table 1).

#### Laboratory parameters (table 1)

Data on serum CRP value, ESR, WBC count, and serum creatinine value on admission was available in 272, 192, 290, and 282 episodes of endocarditis, respectively. At all time points, mortality was significantly higher among the patients with CRP values ≥100 mg/l on admission than among those with lower CRP values. The patients with serum creatinine values ≥100 μmol/l on admission had a significantly higher mortality at 3 months, but not later, than the patients with normal serum creatinine values. The ESR (≥50 mm/h) or WBC counts (≥10 × 10^9^/l) on admission did not associate with mortality (all p values > 0.188).

### Valve surgery

#### Patient characteristics

Of the 109 cardiac operations performed within 1 year after diagnosis, 43 were done within 2 weeks, 16 between 2 and 4 weeks, and 35 between 1 and 3 months. The cumulative proportion of the patients requiring surgical treatment is presented in table 2. The requirement for surgery was at all time points significantly higher in men and in patients < 64 years of age. The need for surgery was unrelated to the causative microorganism. The proportion of the episodes requiring surgical treatment was highest for cases where 2 native valves were infected, with a significant difference between the involvement of 2 valves and all other valve sites as a group (all p values < 0.01).

The requirement for surgery was significantly associated with heart failure and the presence of a major criterion or vegetation on echocardiography. The need for surgery was not different in the patients with background conditions such as diabetes, collagenosis, or malignancy (all p values > 0.080). Patients with IVDU required surgery after 6 months to 1 year significantly less often than those with no IVDU.

#### Laboratory parameters (table 2)

There was a trend for a more common requirement for surgery during the index hospitalisation in patients with CRP values ≥100 mg/l on admission as compared to those with lower CRP values, but the difference did not reach statistical significance (p = 0.060). Patients with ESR ≥50 mm/h on admission required surgery significantly less often than those with lower ESR values at all time points. Elevated levels of the WBC count (≥10 × 10^9^/l) or serum creatinine (≥100 μmol/l) on admission were not significantly associated with the need for surgery.

### Complications during the index hospitalisation

The association between the clinical characteristics and the development of in-hospital complications of infective endocarditis are shown in table 3. Of the 86 patients who had neurological complications, the complication was manifested already on admission in 68.6% (59/86), within 1 week from admission in 76.7% (66/86), and within 2 weeks of admission in 86.1% (74/86) of the patients. There were significant differences in the frequency of neurological complications between infective endocarditis caused by different pathogens, these complications being most common in episodes caused by *S. pneumoniae *and least common in blood culture-negative endocarditis. Neurological complications were most common in infections of 2 native valves, and least common if only the tricuspid valve was infected, but the differences between infections of various valve sites were not significant. There was no difference in the frequency of all neurological complications or major cerebral emboli between the episodes with or without a vegetation, or with or without a major criterion, detected on echocardiography.

Of the 102 patients who had peripheral emboli, the complication was manifested already on admission in 55.9% (57/102), within 1 week from admission in 82.4% (84/102), and within 2 weeks from admission in 88.2% (90/102). Significant differences in the development of peripheral emboli were also observed between various causative agents and affected valve types (p < 0.001). Peripheral emboli were most common in episodes caused by *S. aureus*, infections of 2 native valves and the tricuspid valve, and least common in episodes caused by coagulase-negative staphylococci and in PVE. The occurrence of peripheral emboli was significantly associated with a vegetation detected on echocardiography, and with IVDU.

There were significant differences in the development of heart failure between the various microorganisms as causative agents and between the infected valve sites. Heart failure was most common in episodes caused by *S. pneumoniae *and in infection of 2 native valves, and least common in episodes caused by viridans streptococci and in tricuspid valve endocarditis. Heart failure was significantly associated with the age ≥65 years.

### Factors predicting mortality

#### Patient characteristics

The factors predicting in-hospital and 1-year mortality are presented in table 4. Age < 64 years significantly predicted a favourable outcome within 1 year from admission but not during the index hospitalisation. Infection of 2 native valves significantly predicted both in-hospital and 1-year mortality. The presence of a major criterion or vegetation on echocardiography significantly predicted mortality within 1 year from admission, but not during the index hospitalisation. The occurrence of neurological complications, peripheral emboli, or heart failure significantly predicted both in-hospital and 1-year mortality.

#### Laboratory parameters

There was a significant trend between the level of CRP on admission and both short-term and 1-year outcome (table 4). In the trend analysis of association, it was found that an increment of 50 mg/l of CRP on admission was associated with a 1.33-fold hazard for in-hospital death (HR 1.33, 95% CI 1.06 to 1.68; p = 0.015) and a 1.24-fold hazard for death within 1 year from admission (HR 1.24, 95% CI 1.06 to 1.46; p = 0.009). In a similar analysis, corresponding to every increment of 100 mg/L on admission, the hazard for in-hospital death was 1.78-fold (HR 1.78, 95% CI 1.12 to 2.82; p = 0.015), and the hazard for 1-year death was 1.54-fold (HR 1.54, 95% CI 1.12 to 2.12; p = 0.009). ESR level ≥50 mm/h, WBC count ≥10 × 10^9^/l, or serum creatinine value ≥100 μmol/l on admission did not significantly predict in-hospital or 1-year mortality (table 4).

Among the surgically treated patients, the hazard for in-hospital death for the patients with CRP values ≥100 mg/l on admission was 6.85-fold as compared to those with CRP values < 100 mg/l on admission (HR 6.85, 95% CI 1.51 to 30.95; p = 0.013). Among the conservatively treated patients, no significant association was observed between in-hospital mortality and the level of CRP on admission (p = 0.181). When analysed separately for the surgically and conservatively treated patients, no association was observed between the level of ESR on admission and in-hospital mortality (p values > 0.468).

### Factors predicting requirement for surgery

#### Patient characteristics

The factors predicting the requirement for in-hospital and 1-year surgery are presented in table 5. Male sex and age < 64 years significantly predicted a need for surgery at both of these time points, as did the development of heart failure or the presence of a major criterion or vegetation on echocardiography. Peripheral emboli predicted a need for in-hospital surgery, while *S. pneumoniae *as the causative agent or infection of 2 native valves predicted a need for surgery within 1 year from admission.

#### Laboratory parameters

Patients with CRP values ≥100 mg/l on admission tended to require surgery during the index hospitalisation more commonly than those with lower CRP values, but the difference was not significant (p = 0.063). In the patients who had ESR values ≥50 mm/h on admission, the hazard for in-hospital and 1-year surgery decreased to less than 50% as compared to those with lower ESR values (table 5).

## Discussion

Previous studies have defined various clinical and laboratory findings, which have prognostic significance in patients treated for infective endocarditis [5,8-12,18]. These studies have shown that the outcome of endocarditis may be associated with a number of clinical variables, e.g. age or underlying diseases of the patient, development of complications, echocardiographic findings, laboratory parameters of inflammation, and the virulence of the causative microorganisms. However, some inconsistencies between the results from different hospitals are evident. At least to some extent, this may be due to differences between the institutions or patient populations studied, or even between environmental or genetic factors. We sought to define the prognostic significance of some of the conventional risk predictors in patients treated for infective endocarditis in a Finnish teaching hospital.

Of the laboratory parameters of inflammation, the level of CRP on admission was most prominently associated with the outcome of endocarditis throughout the study period, the prognosis being significantly worse in the patients with high CRP values. Illustratively, in the patients who had CRP values ≥100 mg/l on admission, the hazard ratio for in-hospital death was 2.9-fold and the hazard ratio for 1-year death was 3.9-fold as compared to the patients with lower CRP values (table 4). We are not aware of any previous study focusing on the level of the first CRP value as a prognostic sign in infective endocarditis, although Wallace *et al. *[10] have shown that in-hospital and 6-month mortalities were not affected by an abnormal or normal CRP value within 48 hours of admission. Instead, these authors found that mortality was strongly associated with abnormal WBC counts or serum creatinine concentrations. In our patients, elevated WBC counts on admission did not predict a poor outcome, but there was a tendency for a higher mortality in the patients with elevated serum creatinine concentrations. This probably reflected the worse general condition in these patients.

In many previous studies, *S. aureus *as the causative agent has been associated with an adverse outcome [9,11,19-23]. For example, in a recent study of Cabell *et al. *[9], *S. aureus *endocarditis had a 1.5-fold increase in the risk of death over 1 year as compared to the patients with endocarditis due to other pathogens. Also in the present study, there was a tendency for a higher mortality in *S. aureus *endocarditis as compared to the rest of the cases excluding *S. pneumoniae *endocarditis, but the association was significant only at 3 months. In another recent study [10], *S. aureus *did not confer a worse prognosis than other microorganisms. The authors assumed that this unconventional finding might have been due to the fact that among their patients with *S. aureus *endocarditis there was a high incidence of tricuspid valve involvement in which no deaths occurred. Consistently in our series, 20 of the 75 cases of *S. aureus *endocarditis were in patients with IVDU. Of these patients, 15 had tricuspid valve endocarditis with no mortality. We believe that this may have contributed to the fact that in the present study, the outcome of *S. aureus *endocarditis was not significantly worse than the outcome of endocarditis caused by other microorganisms. The high mortality rate of 45.5% in our patients with pneumococcal endocarditis is in line with previous findings [24], demonstrating the aggressive and destructive course of this disease.

Due to conflicting results of even the recent studies, the relation between survival and echocardiographic findings in infective endocarditis remains controversial. In their comprehensive study on risk classification for mortality, Hasbun *et al. *[11] found that the presence of a vegetation was not associated with increased 6-month mortality of endocarditis. Correspondingly, in a recent series of Chu *et al. *[12] echocardiographic findings were not predictive of in-hospital mortality. It is notable that in these two studies, the vegetation size and mobility were not analysed. Quite the opposite, there are many other studies showing that certain echocardiographic findings are significantly associated with mortality. In a prospective multicentre study, Thuny *et al. *[18] found that vegetation length was a strong predictor of 1-year mortality. When studying patients with aortic or mitral valve endocarditis, Cabell *et al. *[25] showed that vegetation size was a predictor of mortality at 30 days and 1 year. Further, in right-sided endocarditis in drug users, size of vegetation >2 cm proved a major prognostic factor of in-hospital mortality [26]. In another recent series [10], a visible vegetation on echocardiography significantly influenced 6-month mortality, but not in-hospital mortality. Our results are in good correlation with many of these findings, since at 3 and 6 months, and 1 year, mortality of the patients with a vegetation on echocardiography was significantly higher than mortality of the patients without a vegetation. A visible vegetation increased the hazard for death within 1 year to 2.6-fold (table 4). Regrettably, the size and mobility of vegetations were not recorded in the present study.

The role of echocardiography in predicting embolic events has also been disputable, although recent studies suggest that vegetations, and especially certain characteristics of vegetations, are associated with a greater stroke rate [25]. In this respect, our results are different, since a visible vegetation on echocardiography significantly predicted only peripheral emboli, not cerebral emboli or other neurological complications.

In our patients, heart failure was the complication, which was most significantly associated with an adverse outcome during the index hospitalisation and up to 1 year from admission. This corroborates other studies, which have found heart failure to be a major risk factor for mortality in endocarditis [11,27]. Also neurological complications and peripheral emboli significantly predicted both in-hospital and 1-year mortality. These findings compare with many previous reports [5,12]. Among the underlying diseases, diabetes has been shown to be associated with a higher mortality rate [12]. The results of the present study do not corroborate this finding, as mortality in our patients was not dependent of diabetes, or of any other underlying conditions assessed.

Assessment of the role of cardiac surgery as a prognostic factor is very complicated. Patients may not be operated on because they are critically ill while some patients undergo cardiac surgery only because they have large vegetations. In the present study, mortality was somewhat higher for the surgically treated patients than for the patients treated conservatively. This finding is evidently due to a more severe disease with valve destructions in the patients who underwent surgery. The severity of the disease in these surgically treated patients is illustrated e.g. by their having a higher frequency of complications of endocarditis. The association between the requirement for surgery and heart failure was highly significant and, in addition, peripheral emboli were more common in patients who needed surgical treatment, although the association was significant only at the time point of 3 months. Also, these patients had a somewhat higher frequency of neurological complications than those treated conservatively. It is of note that all of these complications were shown to significantly predict mortality in our patients.

Although infective endocarditis is an uncommon disease, the long study period of 25 years made it possible for us to include a considerable number of patients with endocarditis. It is of concern, however, that during such a long period of time, there may have been changes in several aspects of diagnostic and therapeutic management of endocarditis. This is evidently one limitation of the present study.

## Conclusion

Some of the factors (e.g. heart failure, neurological complications, peripheral emboli) predicting a poor prognosis and/or need for surgery in association with endocarditis were the same as observed in previous studies, while some other previously established poor prognostic factors (diabetes, elevated WBC counts) were not present in our patient population. A new finding was that high CRP values on admission significantly predicted both short-term and 1-year mortality in endocarditis.

## Competing interests

The author(s) declare that they have no competing interests.

## Authors' contributions

All of the authors contributed substantially to the study. MH collected the data. MH and PK designed the study and wrote the original version of the manuscript. HH, SM, TS, EE and JN contributed to evaluation of data and provided critical comments for the manuscript. HH and SM performed the statistical analyses. All authors read and approved the final version of the manuscript.

## Pre-publication history

The pre-publication history for this paper can be accessed here:



## References

[B1] Nissen H, Nielsen PF, Frederiksen M, Helleberg C, Nielsen JS (1992). Native valve infective endocarditis in the general population: a 10-year survey of the clinical picture during the 1980s. Eur Heart J.

[B2] Van der Meer JTM, Thompson J, Valkenburg HA, Michel MF (1992). Epidemiology of bacterial endocarditis in the Netherlands. I. Patient characteristics. Arch Intern Med.

[B3] Watanakunakorn C, Burkert T (1993). Infective endocarditis at a large community teaching hospital, 1980–1990. A review of 210 episodes. Medicine (Baltimore).

[B4] Delahaye F, Goulet V, Lacassin F, Ecochard R, Selton-Suty C, Hoen B, Etienne J, Briançon S, Leport C (1995). Characteristics of infective endocarditis in France 1991. A 1-year survey. Eur Heart J.

[B5] Netzer ROM, Zollinger E, Seiler C, Cerny A (2000). Infective endocarditis: clinical spectrum, presentation and outcome. An analysis of 212 cases 1980–1995. Heart.

[B6] Hoen B, Alla F, Selton-Suty C, Béguinot I, Bouvet A, Briançon S, Casalta JP, Danchin N, Delahaye F, Etienne J, Le Moing V, Leport C, Mainardi JL, Ruimy R, Vandenesch F (2002). Changing profile of infective endocarditis. Results of a 1-year survey in France. JAMA.

[B7] Tornos MP, Olona M, Permanyer-Miralda G, Almirante B, Evangelista A, Soler-Soler J (1995). Is the clinical spectrum and prognosis of native valve infective endocarditis in non-addicts changing?. Eur Heart J.

[B8] Delahaye F, Ecochard R, de Gevigney G, Barjhoux C, Malquarti V, Saradarian W, Delaye J (1995). The long term prognosis of infective endocarditis. Eur Heart J.

[B9] Cabell CH, Jollis JG, Peterson GE, Corey GR, Anderson DJ, Sexton DJ, Woods CW, Reller LB, Ryan T, Fowler VG (2002). Changing patient characteristics and the effect on mortality in endocarditis. Arch Intern Med.

[B10] Wallace SM, Walton BI, Kharbanda RK, Hardy R, Wilson AP, Swanton RH (2002). Mortality from infective endocarditis: clinical predictors of outcome. Heart.

[B11] Hasbun R, Vikram HR, Barakat LA, Buenconsejo J, Quagliarello VJ (2003). Complicated left-sided native valve endocarditis in adults. Risk classification for mortality. JAMA.

[B12] Chu VH, Cabell CH, Benjamin DK, Kuniholm EF, Fowler VG, Engemann J, Sexton DJ, Corey GR, Wang A (2004). Early predictors of in-hospital death in infective endocarditis. Circulation.

[B13] Heiro M, Nikoskelainen J, Hartiala JJ, Saraste MK, Kotilainen PM (1998). Diagnosis of infective endocarditis. Sensitivity of the Duke vs von Reyn criteria. Arch Intern Med.

[B14] Heiro M, Nikoskelainen J, Engblom E, Kotilainen E, Marttila RJ, Kotilainen P (2000). Neurologic manifestations of infective endocarditis. A 17-year experience in a teaching hospital in Finland. Arch Intern Med.

[B15] Heiro M, Helenius H, Sundell J, Koskinen P, Engblom E, Nikoskelainen J, Kotilainen P (2005). Utility of serum C-reactive protein in assessing the outcome of infective endocarditis. Eur Heart J.

[B16] Heiro M, Helenius H, Mäkilä S, Savunen T, Engblom E, Nikoskelainen J, Kotilainen P (2006). Infective endocarditis in a Finnish teaching hospital. A study on 326 episodes treated during 1980–2004. Heart.

[B17] Durack DT, Lukes AS, Bright DK, the Duke Endocarditis Service (1994). New criteria for diagnosis of infective endocarditis: utilization of specific echocardiographic findings. Am J Med.

[B18] Thuny F, Disalvo G, Belliard O, Avierinos JF, Pergola V, Rosenberg V, Casalta JP, Gouvernet J, Derumeaux G, Iarussi D, Ambrosi P, Calabro R, Riberi A, Collart F, Metras D, Lepidi H, Raoult D, Harle JR, Weiller PJ, Cohen A, Habib G (2005). Risk of embolism and death in infective endocarditis: prognostic value of echocardiography. A prospective multicenter study. Circulation.

[B19] Sanabria TJ, Alpert JS, Goldberg R, Pape LA, Cheeseman SH (1990). Increasing frequency of staphylococcal infective endocarditis. Experience at a university hospital, 1981 through 1988. Arch Intern Med.

[B20] Fernandez-Guerrero ML, Verdejo C, Azofra J, Azofra J, de Gorgolas M (1995). Hospital-acquired infectious endocarditis not associated with cardiac surgery: an emerging problem. Clin Infect Dis.

[B21] Røder BL, Wandall DA, Frimodt-Møller N, Espersen F, Skinhoj P, Rosdahl VT (1999). Clinical features of *Staphylococcus aureus *endocarditis: a 10-year experience in Denmark. Arch Intern Med.

[B22] Miro JM, Anguera I, Cabell CH, Chen AY, Stafford JA, Corey GR, Olaison L, Eykyn S, Hoen B, Abrutyn E, Raoult D, Bayer A, Fowler VG, International Collaboration on Endocarditis Merged Database Study Group (2005). *Staphylococcus aureus *native valve infective endocarditis: Report of 566 episodes from the International Collaboration on endocarditis merged database. Clin Infect Dis.

[B23] Nadji G, Rémadi JP, Coviaux F, Ali Mirode A, Brahim A, Enriquez-Sarano M, Tribouilloy C (2005). Comparison of clinical and morphological characteristics of *Staphylococcus aureus *endocarditis with endocarditis caused by other pathogens. Heart.

[B24] Aronin SI, Mukherjee SK, West JC, Cooney EL (1998). Review of pneumococcal endocarditis in adults in the penicillin era. Clin Infect Dis.

[B25] Cabell CH, Pond KK, Peterson GE, Durack DT, Corey GR, Anderson DJ, Ryan T, Lukes AS, Sexton DJ (2001). The risk of stroke and death in patients with aortic and mitral valve endocarditis. Am Heart J.

[B26] Martin-Dávila P, Navas E, Fortún J, Moya JL, Cobo J, Pintado V, Quereda C, Jimenez-Mena M, Moreno S (2005). Analysis of mortality and risk factors associated with native valve endocarditis in drug users: The importance of vegetation size. Am Heart J.

[B27] Siddiq S, Missri J, Silverman DI (1996). Endocarditis in an urban hospital in the 1990s. Arch Intern Med.

